# Patient-Reported Outcomes in ATLAS and FLAIR Participants on Long-Acting Regimens of Cabotegravir and Rilpivirine Over 48 Weeks

**DOI:** 10.1007/s10461-020-02929-8

**Published:** 2020-05-23

**Authors:** Miranda Murray, Antonio Antela, Anthony Mills, Jenny Huang, Hans Jäger, Enrique Bernal, Johan Lombaard, Harold Katner, Sharon Walmsley, Marie-Aude Khuong-Josses, Krischan Hudson, David Dorey, Sandy Griffith, William Spreen, Simon Vanveggel, Mark Shaefer, David Margolis, Vasiliki Chounta

**Affiliations:** 1 Health Analytics and Outcomes Ltd, London, UK; 2 Hospital Clínico Universitario, Santiago de Compostela, Spain; 3 Southern California Men’s Medical Group, West Hollywood, CA USA; 4 GlaxoSmithKline, Mississauga, ON Canada; 5 MUC Research GmbH, Munich, Germany; 6 MVZ Karlsplatz, HIV Research and Clinical Care Centre, Munich, Germany; 7 Hospital General Universitario Reina Sofía, Murcia, Spain; 8 Josha Research, Bloemfontein, South Africa; 9 Mercer University Medical School, Macon, GA USA; 10 University Health Network, Toronto, ON Canada; 11 Hôpital Delafontaine, Saint-Denis, France; 12 ViiV Healthcare, Research Triangle Park, NC USA; 13 Janssen Research & Development, Beerse, Belgium; 14 ViiV Healthcare, 980 Great West Road, Brentford, TW8 9GS Middlesex UK

**Keywords:** Patient-reported outcomes, Antiretroviral therapy, Cabotegravir, Rilpivirine, Long-acting treatment

## Abstract

The phase 3 ATLAS and FLAIR studies demonstrated that maintenance with Long-Acting (LA) intramuscular cabotegravir and rilpivirine is non-inferior in efficacy to current antiretroviral (CAR) oral therapy. Both studies utilized Patient-Reported Outcome instruments to measure treatment satisfaction (HIVTSQ) and acceptance (ACCEPT general domain), health status (SF-12), injection tolerability/acceptance (PIN), and treatment preference. In pooled analyses, LA-treated patients (n = 591) demonstrated greater mean improvements from baseline than the CAR group (n = 591) in treatment satisfaction (Week 44, + 3.9 vs. +0.5 HIVTSQs-points; *p* < 0.001) and acceptance (Week 48, +8.8 vs. +2.0 ACCEPT-points; *p* < 0.001). The acceptability of injection site reactions (PIN) significantly improved from week 5 (2.10 points) to week 48 (1.62 points; *p* < 0.001). In both studies, ≥ 97% of LA group participants with recorded data preferred LA treatment compared with prior oral therapy. These results further support the potential of a monthly injectable option for people living with HIV seeking an alternative to daily oral treatment.

**Electronic supplementary material:**

The online version of this article (10.1007/s10461-020-02929-8) contains supplementary material, which is available to authorized users.

## Introduction

Combination antiretroviral therapy (cART) has dramatically reduced mortality and improved the quality of life of people living with HIV (PLWHIV) [1, 2], but HIV treatment currently requires a lifelong commitment to daily oral therapy. Emotional and adherence-related challenges associated with daily cART impact many PLWHIV which in turn has led to significant interest in antiretroviral therapy (ART) with less frequent dosing [3–9]. As a result, a clinical development program has been dedicated to introducing a long-acting (LA) alternative to daily oral cART, providing an additional treatment option that may improve treatment adherence and satisfaction for PLWHIV.

LA injectable formulations have been developed for cabotegravir (CAB), an integrase strand transfer inhibitor (INSTI), and rilpivirine (RPV), a non-nucleoside reverse transcriptase inhibitor (NNRTI) [10, 11]. It has been shown in both individual and pooled analyses from two pivotal phase 3 studies in treatment-experienced patients (ATLAS; NCT02951052) and in previously treatment-naïve patients (FLAIR; NCT02938520) that monthly dosing of CAB+RPV LA is non-inferior to daily oral ART for maintaining HIV suppression [12–14]. In addition, other than injection-site reactions (ISRs), which declined over time, the overall incidence of adverse events was comparable between the oral and LA treatment groups [12–14].

Similarly, in the phase 2b LATTE-2 study (NCT02120352), CAB+RPV LA maintenance every 1 or 2 months resulted in similar rates of virologic suppression to continuing on the oral induction regimen of oral CAB with nucleoside analogues through 96 weeks [15]. In qualitative interviews to assess their preference for LA injections over oral medications, LATTE-2 participants in Spain and the US cited both the convenience of LA injections vs. daily pills, and psychosocial benefits of LA treatment such as confidentiality, the lack of a daily reminder of living with HIV, and the reduced risk of stigma associated with disease disclosure as reasons supporting their preference for an LA therapy [16].

Patient-reported outcomes (PROs) are important for the holistic assessment of new medicines and innovative treatment options. In ATLAS, PROs were assessed in participants who had been virologically suppressed (HIV-1 RNA < 50 c/mL) on oral regimens for a median of 4 years, allowing participants to compare their experiences of LA treatment against prior oral therapies. In contrast, the assessment of PROs in FLAIR presented an opportunity to assess switching to the LA treatment vs. remaining on first-line oral treatment in patients who were treatment-naïve prior to study enrollment. Both studies assessed patient satisfaction with, and acceptance of, the oral or LA treatment received; the acceptability and tolerability of CAB+RPV LA injections, and overall patient health status. Treatment preference and reason for wishing to switch from oral to LA therapy were assessed as exploratory endpoints. Herein, we present both pooled and individual PRO data from the ATLAS and FLAIR studies.

## Methods

### Study Design

**Fig. 1:**
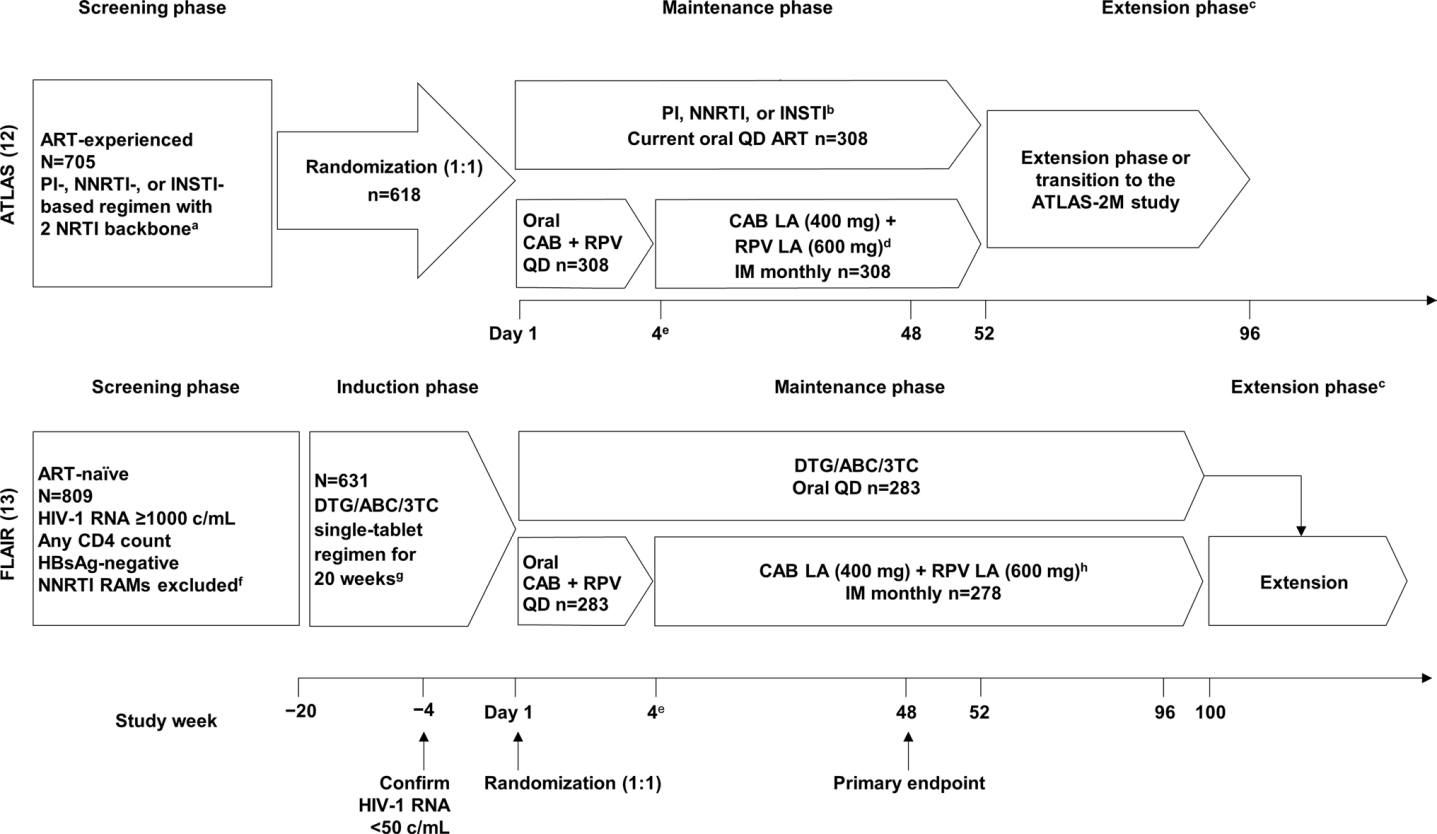
The ATLAS [12] and FLAIR [13] study design. Eligible individuals were randomly assigned (1:1) to continue their current antiretroviral regimen (CAR arm) or switch to the long-acting regimen (LA arm). Those assigned to the LA arm initially received 4 weeks of oral CAB+RPV QD, then transitioned to the injectable regimen. ^a^Uninterrupted ART for 6 months and VL < 50 c/mL at screening, 2 × VL < 50 c/mL for ≤ 12 months. ^b^INSTI-based regimen capped at 40% of enrollment; Triumeq excluded from study. ^c^Optional switch to CAB+RPV LA at week 52 for those on CAR. ^d^Participants who withdraw/complete CAB+RPV LA must complete 52 weeks of follow-up. ^e^Participants received an initial loading dose of CAB LA (600 mg) and RPV LA (900 mg) at week 4b. From week 8 onwards, participants received CAB LA (400 mg)+RPV LA (600 mg) injections every 4 weeks. ^f^NNRTI RAMs but not K103N were exclusionary. ^g^Of the 631 participants who entered the induction phase, two withdrew prior to receiving study drug. DTG plus two alternative non-ABC NRTIs was permitted if participant was intolerant or HLA-B*5701-positive (n = 30 as last regimen during induction: n = 2 discontinued during induction, n = 14 randomized to CAB+RPV LA, n = 14 randomized to DTG/ABC/3TC arm and continued on DTG plus two alternative non-ABC NRTIs in the maintenance phase). ^h^Participants who withdraw/complete CAB+RPV LA enter 52-week long-term follow-up. *3TC* lamivudine, *ABC* abacavir, *ART* antiretroviral therapy, *CAB* cabotegravir, *CAR* current antiretroviral regimen, *DTG* dolutegravir, *IM* intramuscular, *INSTI* integrase strand transfer inhibitor, *HBsAg* hepatitis B surface antigen, *LA* long-acting, *NNRTI* non-nucleoside reverse transcriptase inhibitor, *NRTI* nucleoside reverse transcriptase inhibitor, *PI* protease inhibitor, *RAM* resistance-associated mutation, *RPV* rilpivirine, *VL* viral load

ATLAS and FLAIR (Fig. 1) are ongoing, phase 3, randomized, open-label, parallel-group studies comparing the efficacy and safety of monthly intramuscular (IM) CAB+RPV LA (400 mg CAB+600 mg RPV) vs. continuation of an oral cART regimen—the current antiretroviral regimen (CAR) group—consisting of either a pre-existing stable daily oral therapy of at least 6 months’ prior duration (ATLAS) or a defined oral induction treatment comprising a single-tablet coformulation of dolutegravir, abacavir, and lamivudine (FLAIR). Participant recruitment and study designs have been fully described in the primary clinical manuscripts [12, 13].

Both studies were conducted in accordance with the principles founded in the Declaration of Helsinki and with Good Clinical Practice. All participants provided written informed consent, and the protocol was approved by an institutional review board or ethics committee of each study site. The authors can attest for adherence to the study protocol and for the accuracy and completeness of the data and analyses.

### Assessments and Endpoints

PRO instruments (Table 1) were included at specific timepoints in each study to assess health status, treatment satisfaction, acceptance and preference, and the tolerability and acceptability of injections. These instruments were selected based on qualitative interviews and PRO data from patients enrolled on the phase 2b LATTE-2 study [16].

**Table 1:** The PRO instruments conducted in ATLAS and FLAIR

PRO instrument	Assessment	Timepoints measured	Pooled analysis
HIVTSQs/c^a^	Patient satisfaction with HIV treatment	Status version: maintenance baseline, week 4, week 24, week 44 Change version: week 48	✔
ACCEPT	Patient acceptance of treatment	Maintenance baseline, week 8, week 24, week 48	✔
PIN questionnaire	Perception of pain and injection site reactions	Week 5, week 41, week 48	✔
SF-12	General health status and degree of mental health distress	Maintenance baseline, week 24, week 48	✖
Preference of HIV treatment (single question)	Patient preference for CAB + RPV LA vs. their current oral therapy	Week 48	✖
Reason for switch (single question)	Patient reasoning for switching to LA therapy from oral therapy for ATLAS study only	Week 52	✖
HAT-QoL^b^	Overall function and wellbeing. Only 3 out of 9 dimensions were assessed	Maintenance baseline, week 24, week 48	✖
Numeric Rating Scale^c^	Intensity of post-injection pain	Week 4, week 5, week 40, week 41	✖

*ACCEPT* Chronic Treatment Acceptance questionnaire, *CAB* cabotegravir, *HAT-QoL* HIV/AIDS-targeted quality of life, *HIVTSQs/c* HIV Treatment Satisfaction Questionnaire (status version)/(change version), *LA* long-acting, *PIN* Perception of Injection questionnaire, *PRO* patient-reported outcome, *RPV* rilpivirine, *SF-12* 12-Item Short Form Health Survey

^a^HIVTSQc only administered to participants receiving LA therapy

^b^No meaningful differences between arms were reported with the three dimensions included in the phase 3 studies. Results are not discussed here

^c^Consistent with the PIN, numerical reduction in post-injection pain was reported in the Numeric Rating Scale over time. No significance testing was preplanned for this measure. Results are not discussed here

The PRO instruments used in ATLAS and FLAIR included:

#### Treatment Satisfaction: HIVTSQ

Treatment satisfaction was assessed using a recent adaptation of the validated 10-item HIV Treatment Satisfaction Questionnaire (HIVTSQ) [17, 18], which included two additional items to account for LA dosing [19, 20], specifically:

11. *How easy or difficult have you been finding your treatment to be recently?*
12. *How satisfied are you with the amount of discomfort or pain involved with your present form of treatment?*

Extensive psychometric analyses on the 12-item HIVTSQ suggest that these two additional items do not reduce the overall validity of the questionnaire [19, 20]. Item 11 contributes to the structure of the scale, improving the outcomes of confirmatory factor analysis, and preserves the option of calculating the total score with items 1 through 11. Item 12 can be reported individually and is not included in the total score [19–21]. Two versions exist of the HIVTSQ: the status version (HIVTSQs), which was the first to be developed, and the subsequent change version (HIVTSQc) developed to mitigate ceiling effects common in treatment satisfaction measures [18]. The HIVTSQs asked patients to rank their response on a 6-point Likert scale, from 6 (very satisfied) to 0 (very dissatisfied), while the HIVTSQc asked patients to rank their response from 3 (much more satisfied now) to − 3 (much less satisfied now) [18]. On both versions, the scores are added together, to provide the total summary score. The HIVTSQs was assessed at maintenance baseline (MBL) and weeks 4, 24, and 44 in the LA treatment group and the CAR group in both studies, while the HIVTSQc was applied only at week 48 to both treatment groups in FLAIR and to the LA group only in ATLAS.

#### Treatment Acceptance: ACCEPT Questionnaire

Participants in both treatment groups completed the General Acceptance domain of the Chronic Treatment Acceptance (ACCEPT) questionnaire [22], a generic medication acceptance instrument validated for chronic conditions that was administered at MBL and weeks 8, 24, and 48. This domain consists of three questions:

- *Do you agree with the following statement: “My medication has more advantages than disadvantages”?*
- *Given the advantages and disadvantages of your medication, do you consider it to be an acceptable solution?*
- *Are you convinced that in the long term, it is worth taking your medications?*

Responses were rated on a 5-point Likert scale with a score of 5 representing “I don’t know” for all three questions, and scores of 1 through 4 representing increasing levels of agreement or acceptance from “Totally disagree/Not at all acceptable/Not at all convinced” through to “Totally agree/Totally acceptable/Totally convinced”. Item scores were grouped together to form an aggregate that was linearly transformed to range from 0 to 100, with a higher score suggesting greater acceptance [23].

#### Injection Acceptability and Tolerability: PIN Questionnaire

The acceptability and tolerability of monthly injections and ISRs at early (week 5) and later stages of treatment (weeks 41 and 48) was assessed in the LA treatment groups of both studies using the Perception of Injection (PIN) questionnaire. This instrument was adapted for gluteal IM administration from the earlier Vaccinees’ Perception of Injection (VAPI) questionnaire [24], while retaining the same scoring system. The questionnaire contains 21 items in total and consists of four dimensions: “acceptance of ISRs”, “bother of ISRs”, “sleep”, and “leg movement”, and five individually reported items related to anxiety before and after the injection, willingness to receive an HIV injectable treatment at the following visit, pain during injection, and satisfaction with the mode of treatment administration. Participants were asked to score their responses from 1 to 5, where scores of 1 through 3 refer to, in order, “totally acceptable”, “very acceptable”, and “moderately acceptable”; a score of 4 is “a little acceptable”, and 5 is “not at all acceptable”. To avoid multiplicity issues, hypothesis testing was preplanned only for the “acceptance of ISRs” dimension of the PIN.

#### General Health Status: SF-12

General health was assessed in both studies using the generic validated 12-Item Short Form Health Survey (SF-12) questionnaire [25–28]. The SF-12 includes the same eight domains as the older SF-36 questionnaire [29], but with fewer questions, making it more practical, especially for larger and more complex populations [30]. The physical component score (PCS) and mental component score (MCS) of the SF-12 are norm-based and range from 0 to 100, with higher scores indicating better health. In the US population, the mean SF-12 score is 50 with a standard deviation of 10 [31]. SF-12 was administered at MBL, week 24, and week 48 to participants on both treatment arms.

#### Preference Question

For both studies, a single-item preference question was developed to assess participants’ preference for CAB+RPV LA compared with the oral cART received prior to randomization. This was administered, at week 48, to participants in the LA arm only. The question reads: *“For the past 44 weeks you have received Long Acting injectable HIV medication every month. Today we would like you to compare your experience on the Long Acting injections with the oral medication you received during the induction phase of the study. Which therapy do you prefer?”* Participants had the option of selecting either daily oral treatment or monthly injections.

#### Reason for Switch

Additionally, in the ATLAS study only, a single-question “Reason for switch” questionnaire with six response options was administered to all participants (including those continuing on oral CAR) on day 1 to explore the reasons why participants wished to enter an LA trial. A similar questionnaire was also administered at week 52 to participants in the oral CAR arm who had opted to switch to CAB+RPV LA after the primary analysis at week 48, under the study protocol.

### Statistical Analysis

Descriptive statistics summarize questionnaire scores for each timepoint. Statistical comparisons between treatment groups in change from MBL for prespecified endpoints was performed with an ANCOVA model adjusting for covariates selected a priori: MBL score, sex at birth, age, race (white, non-white), third agent class (integrase inhibitors, protease inhibitors, NNRTI) for ATLAS only, and induction baseline HIV RNA for FLAIR only. *P* values and 95% CI for the treatment difference between groups were reported. Within-group comparisons in change over time for the “Acceptance of ISRs” dimension of the PIN was based on the Wilcoxon signed-rank test. The significance threshold was set at 0.05. In addition to individual study analyses, pooled analyses combining data from both ATLAS and FLAIR were performed post hoc for instruments assessing treatment satisfaction, treatment acceptance, and the tolerability of injections and ISRs, adjusting for baseline score, sex at birth, age (< 50, ≥ 50 years) and race (white, non-white). For the exploratory endpoints (preference question and reason for switch) proportions are reported for observed cases without imputation, statistical modeling, or testing. Missing data for non-exploratory PROs were imputed using a last-observation-carried-forward approach including measures assessed at time of withdrawal.

## Results

### Baseline Characteristics

The pooled intention-to-treat-exposed (ITT-E) population of all randomized participants who received at least one dose of study medication in either ATLAS or FLAIR consisted of 1182 individuals: 591 in each treatment group (LA or CAR). MBL characteristics were generally similar between groups in each study and were also similar between the two studies [12, 13], although ATLAS had a numerically higher percentage of participants in the CAR group aged over 50 years compared with the LA group (31% vs. 21%, respectively) [12]. More than 20% of patients enrolled in each study were female, exceeding the recruitment goals for both. Of note, participants enrolled in ATLAS had been on previous cART for a median of 4 years (range 1–21) [12], while participants in FLAIR had no cART experience prior to the induction phase of the study [13].

### PROs with both Individual and Pooled Study Data (HIVTSQ, ACCEPT, PIN)

#### HIVTSQ

Mean HIVTSQs total score values at MBL were high and similar between the oral and LA treatment groups in both ATLAS and FLAIR, with higher mean scores in FLAIR. Out of a maximum of 66 points, mean MBL values in the pooled dataset were 57.1 (SD 8.4) for the CAR group and 57.1 (8.6) for the LA group; while individually these values were, in ATLAS, 55.4 (8.7) and 55.3 (9.1), respectively, and in FLAIR, 59.1 (7.6) and 59.3 (7.4), respectively.

**Fig. 2:**
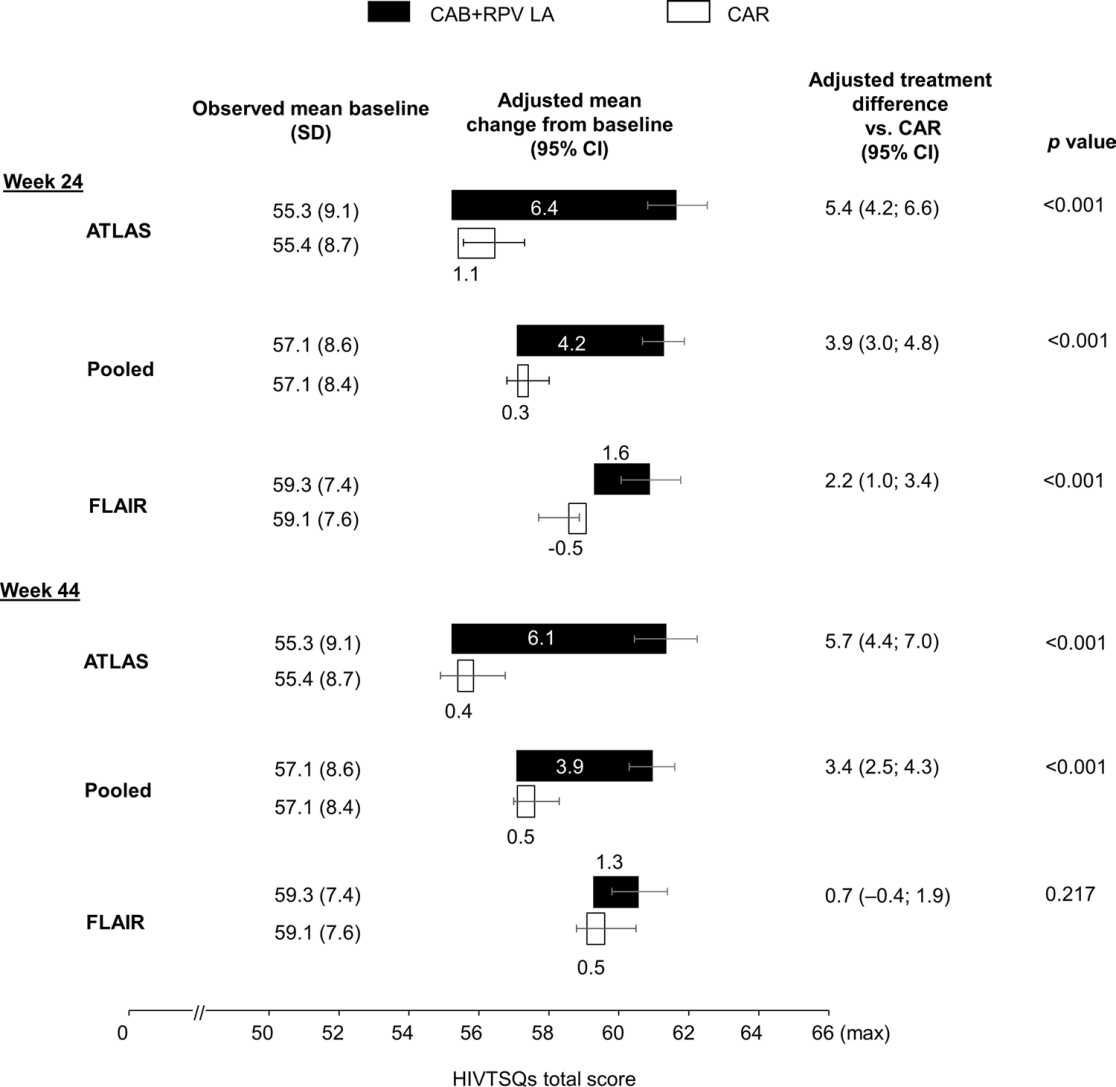
Change from maintenance baseline HIVTSQs total score through week 44. Adjusted mean change from maintenance baseline is estimated from an ANCOVA model. Covariates are: ATLAS: baseline score, sex at birth, age (< 50 vs. ≥ 50 years), race, and third agent class, FLAIR: maintenance baseline score (day 1), sex at birth, age (< 50 vs. ≥ 50 years), race, and induction baseline (week –20) HIV-1 RNA (< 10^5^ vs. ≥ 10^5^ c/mL) Pooled: maintenance baseline score, sex at birth, age (< 50 vs. ≥ 50 years), and race. *CAB* cabotegravir, *CAR* current antiretroviral treatment (oral), *CI* confidence interval, *HIVTSQs* HIV Treatment Satisfaction Questionnaire (status version), *LA* long-acting, *RPV* rilpivirine, *SD* standard deviation

Pooled data at weeks 24 and 44 showed a statistically significant greater improvement in treatment satisfaction from MBL on LA treatment compared with the CAR group (Fig. 2). This difference was mainly driven by ATLAS data, where LA-treated participants showed a large and stable 5.4 (95% CI 4.2–6.6) to 5.7 (95% CI 4.4–7.0)-point treatment difference in the adjusted mean change from MBL in HIVTSQs total score vs. the CAR group at both timepoints that meets the minimum clinically important difference threshold using the distribution-based approach, with the mean difference between the two groups exceeding one half of the standard deviation at baseline [32, 33]. Changes from MBL in ATLAS were greater for LA dosing across all individual items in the HIVTSQs, particularly for items related to overall regimen satisfaction; the ease, convenience, and flexibility of treatment; and satisfaction at the prospect of continuing current treatment (Supplementary Fig. 1).

A smaller LA treatment effect for HIVTSQs total score was reported in the FLAIR study. A statistically significant greater improvement from MBL of 2.2 (95% CI 1.0–3.4) points in the adjusted mean HIVTSQs total score favoring the LA group vs. CAR was observed at week 24 that was not maintained at week 44 (Fig. 2).

Inter-group comparative data for the HIVTSQ change instrument in FLAIR, allowed for a direct comparison of LA maintenance with the oral induction regimen at week 48, with mitigation of ceiling effects caused by high MBL satisfaction. Here, a statistically significant difference (*p* < 0.001) in mean HIVTSQc total score was observed in favor of the LA arm, with an LA adjusted mean of 29.6 (SE 0.49) and a CAR adjusted mean of 25.2 (SE 0.48) giving an adjusted mean treatment difference of 4.1 (95% CI 2.8–5.5).

#### ACCEPT

Mean general acceptance domain scores were high and similar in both studies between the LA and CAR treatment groups at MBL. Pooled mean (SD) MBL values were 80.5 (24.8) for the LA arm and 78.8 (25.3) for the CAR arm, out of a maximum 100 points. In ATLAS, the mean (SD) baseline score was 75.9 (26.5) in the LA group, and 74.7 (26.1) in the CAR group, while in FLAIR these MBL values were 86.0 (21.3) and 83.4 (23.7), respectively.

**Fig. 3:**
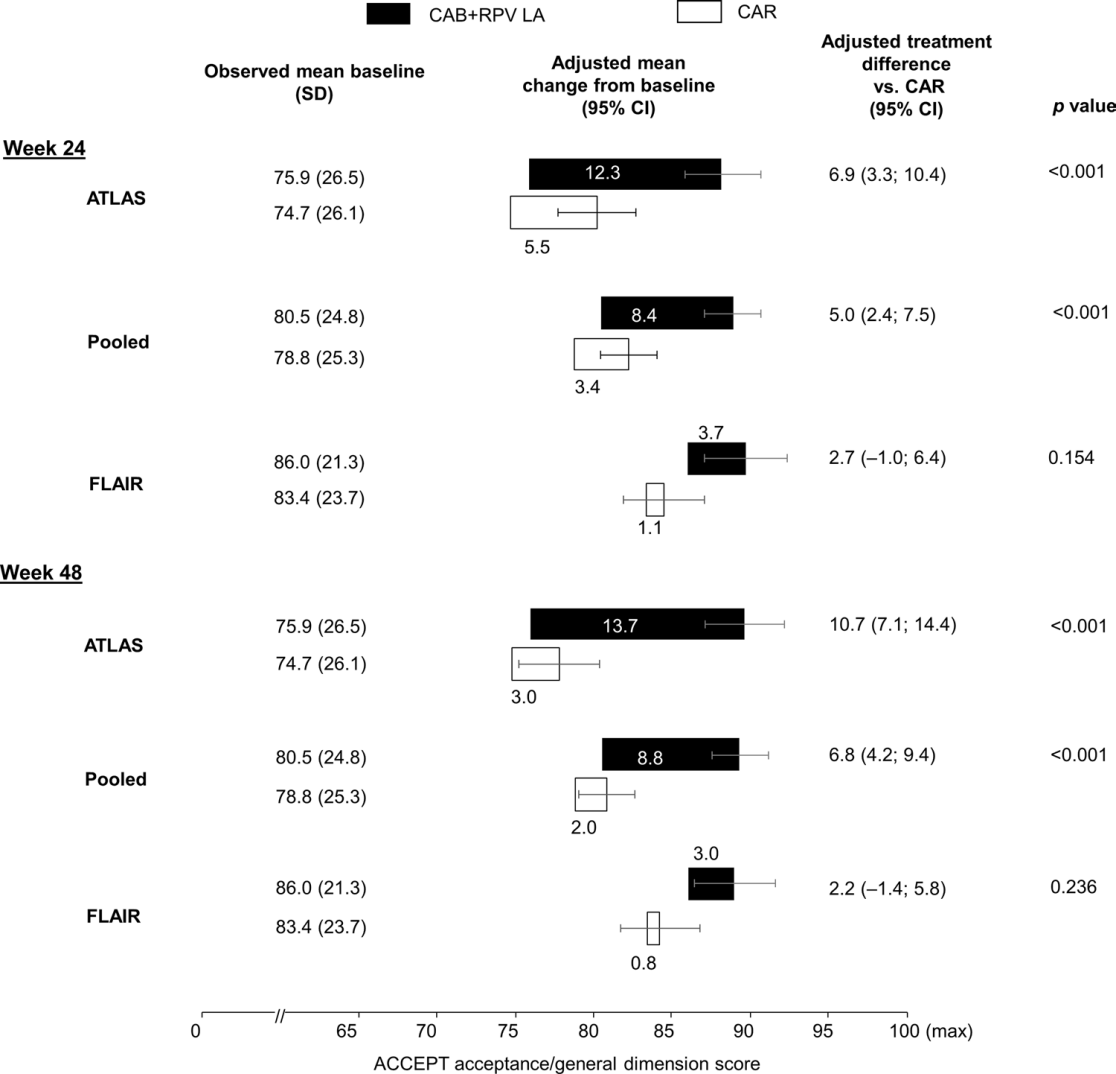
Change from maintenance baseline General Acceptance domain scores through week 48. Adjusted mean change from maintenance baseline is estimated from an ANCOVA model. Covariates are: ATLAS: treatment, baseline score, sex at birth, age (< 50 vs. ≥ 50 years), race, and third agent class, FLAIR: maintenance baseline score (day 1), sex at birth, age (< 50 vs. ≥ 50 years), race, and induction baseline (week –20) HIV-1 RNA (< 10^5^ vs. ≥ 10^5^ c/mL) Pooled: maintenance baseline score, sex at birth, age (< 50 vs. ≥ 50 years), and race. *ACCEPT* Chronic Treatment Acceptance questionnaire, *CAB* cabotegravir, *CAR* current antiretroviral treatment (oral), *CI* confidence interval, *LA* long-acting, *RPV* rilpivirine, *SD* standard deviation

Significantly greater (*p* < 0.001) improvement in the adjusted mean general acceptance domain score change from MBL was noted for LA treatment vs. oral cART in pooled data at weeks 24 and 48 (Fig. 3). Consistent with HIVTSQs data, this improvement was driven largely by ATLAS, in which increases of 16–18% over MBL were seen on LA treatment at weeks 24 and 48 (week 24 adjusted mean change 12.3 [95% CI 9.9–14.8] points; week 48 adjusted mean change 13.7 [95% CI 11.2–16.3] points) compared with lesser changes in the CAR group (week 24 adjusted mean change 5.5 [95% CI 3.0–8.0] points; week 48 adjusted mean change 3.0 [95% CI 0.4–5.6] points). The treatment difference in FLAIR was smaller and non-significant, partly due to the similarly high MBL values noted also in the HIVTSQs.

#### PIN

**Fig. 4:**
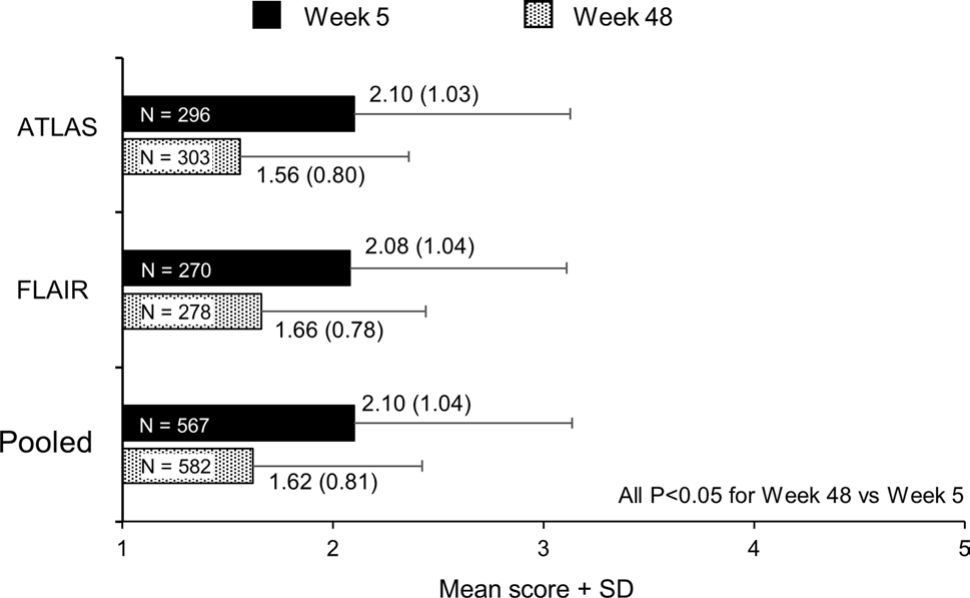
Summary of PIN “Acceptability of ISRs” scores through week 48. Week 48 was compared with the 1st visit (week 5) based on Wilcoxon signed-rank test, respectively. *P* values are derived for ‘Acceptance’ only and not adjusted for multiple testing. *ISR* injection site reaction, *PIN* perception of injection, *SD* standard deviation

According to the “Acceptance of ISRs” dimension of the PIN, most participants in both studies reported that pain and ISRs were “very acceptable” or “totally acceptable” when questioned a week after their first injections of CAB+RPV LA (week 5). In pooled data, the mean (SD) score for the acceptance domain at week 5 was 2.10 (1.04), with favorable scores also reported for the other domains and individual PIN items after the first injection (Supplementary Fig. 2). A statistically significant (*p* < 0.001) mean improvement from week 5 in the acceptance domain was observed in pooled data at weeks 41 (mean 1.67 [SD 0.86]) and 48 (1.62 [0.81]) indicating improved acceptability of ISRs over time, alongside high initial acceptance rates (Fig. 4). For the two items generating the “Acceptance of ISRs” dimension, 90% and 86% of participants in ATLAS and FLAIR, respectively, reported that their ISRs were either “totally acceptable” or “very acceptable” at week 48, while 86% and 84% of participants, respectively, reported that the level of pain experienced was either “totally acceptable” or “very acceptable” at week 48. Consistent results were observed in all remaining domains and individual item scores, although hypothesis testing was not undertaken for other components of the PIN to avoid multiplicity.

### PROs with Individual Study Data Only

#### SF-12

**Fig. 5:**
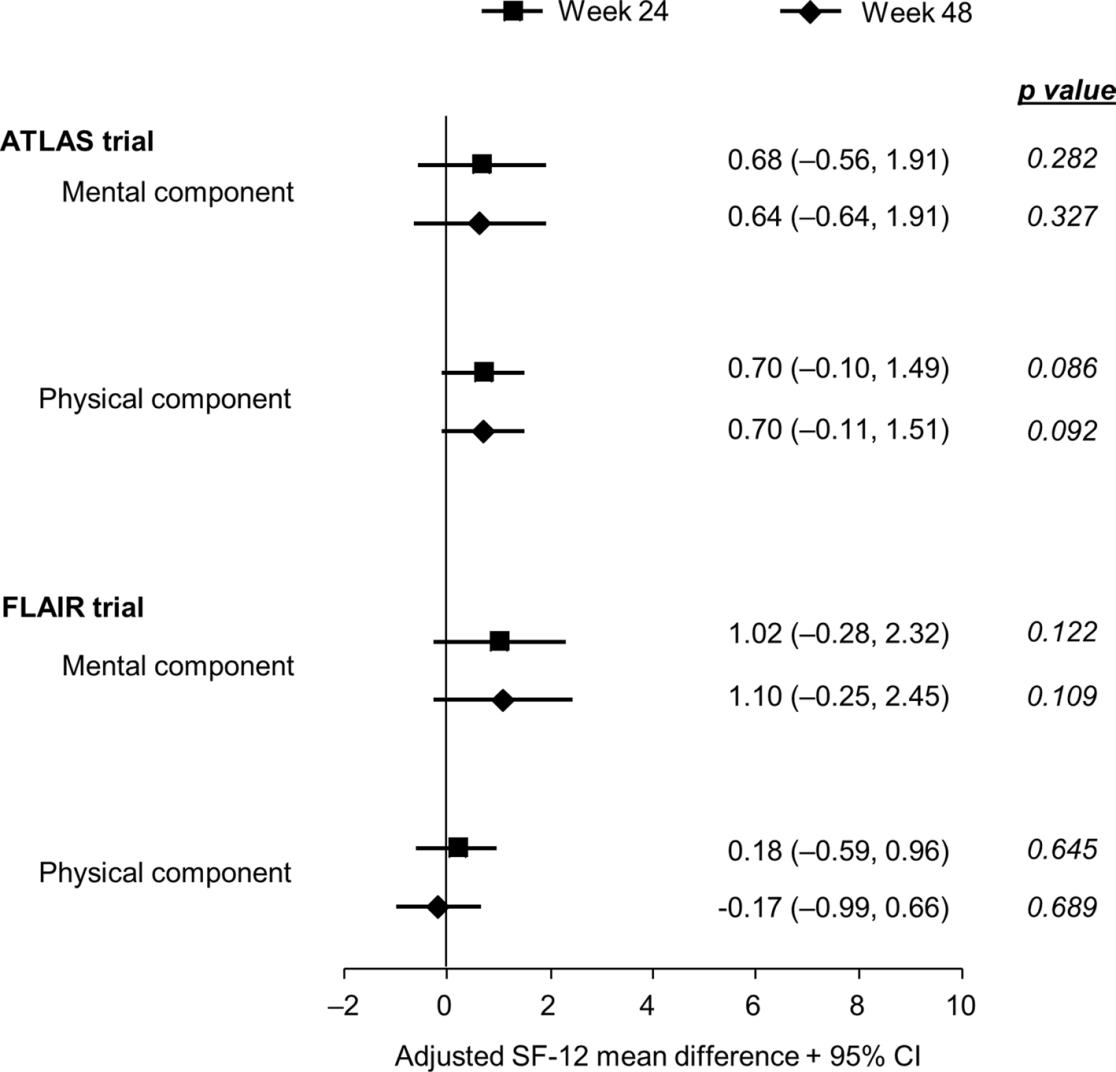
The SF-12 component scores adjusted treatment difference through 48 weeks. Adjusted mean is the estimated mean change from maintenance baseline score by visit in each treatment calculated from an ANCOVA model including the covariates, which are: ATLAS: baseline score, sex at birth, age, race (white, non-white), and third agent class (integrase inhibitors, protease inhibitors, NNRTI), FLAIR: maintenance baseline (day 1) score, induction baseline (week –20) HIV-1 RNA (< 100,000 vs. 100,000 c/mL), sex at birth, age (< 50 vs. ≥ 50 years), and race (white vs. non-white). *CI* confidence interval, *SF-12* 12-Item Short Form Health Survey

No LA vs. CAR difference was observed in either ATLAS or FLAIR for the SF-12 MCS or PCS at MBL or later. In each study, MBL scores were above the US national average of 50 [31] and no significant change from MBL was observed or expected in either domain over 48 weeks. ATLAS patients receiving LA therapy had a mean adjusted treatment difference of 0.64 (95% CI, − 0.64, 1.91; *p* = 0.327) in the MCS domain and 0.70 (95% CI, − 0.11, 1.51; *p* = 0.092) in the PCS domain at 48 weeks. In FLAIR, patients receiving LA therapy had a mean adjusted treatment difference of 1.10 (95% CI [− 0.25, 2.45]; *p* = 0.109) in the MCS domain and − 0.17 (95% CI [− 0.99, 0.66]; *p* = 0.689) in the PCS domain at 48 weeks (Fig. 5).

#### Preference Question (Exploratory)

In the ITT-E population, 86% (266/308) of LA arm participants in ATLAS and 91% (257/283) of LA arm participants in FLAIR rated the LA treatment as their preferred option over daily oral therapy after 48 weeks of treatment. Only 2% (7/308) in ATLAS and 1% (2/283) in FLAIR preferred daily oral treatment, with the remaining 11% (35/308) and 8% (24/283), respectively, classified as missing data. A post hoc observed analysis of participants with response data at week 48 showed that almost all responders preferred the LA regimen over CAR: 97% (266/273) in ATLAS and 99% (257/259) in FLAIR. No participant refused to respond to the preference question.

#### Reason for Switch in ATLAS (Exploratory)

At day 1 (randomization), ATLAS participants indicated “I am interested in research of new therapies” (82%; 505/616) and “My clinician asked me to participate” (25%; 151/616) as the top two reasons for wanting to enter the study and receive an LA regimen. At week 52, those opting to switch from continued oral CAR to open-label CAB+RPV LA in the extension phase indicated that “Interest in the convenience of a monthly injectable treatment” (53%; 164/308) and “Discretion associated with a monthly injectable treatment” (32%; 98/308) were the top two reasons for choosing LA treatment.

## Discussion

Monthly CAB+RPV LA injections have demonstrated similar efficacy vs. daily oral ART in the pivotal phase 3 clinical trials ATLAS and FLAIR [12–14]. Although ISRs were common, they declined over time [12–14] and LA participants reported significantly higher levels of treatment satisfaction and preference for the LA regimen over previous daily oral ART, with respect to baseline, after almost 1 year of follow-up. Discontinuations due to ISRs were very uncommon (1%; 6/489), supporting these findings.

Treatment satisfaction with LA therapy, as assessed by the HIVTSQs instrument, reached similarly high scores in both studies, highlighting very high levels of satisfaction with LA treatment irrespective of prior ART experience. Differences in the change from MBL in treatment satisfaction were observed between ATLAS and FLAIR which could be explained by a difference in baseline satisfaction between the two studies. Changes from MBL in scores for the individual items within the HIVTSQs were higher in the LA group in ATLAS, showing the extent to which LA treatment contributes to improvement across almost all aspects of treatment satisfaction compared with oral therapy. Changes from baseline in FLAIR were generally smaller, particularly in the LA group, due to the ceiling effects caused by very high MBL scores. This observation is consistent with the assumption that the previously treatment-naïve patient group in FLAIR, who were virologically suppressed prior to LA maintenance by a short course of oral treatment with a well-tolerated, single-tablet dolutegravir-based modern regimen, would tend to be inherently more satisfied with their experience of their first oral treatment compared with those in ATLAS, who had already spent a number of years on oral therapies and were better able to compare treatment modalities.

Similarly, treatment acceptance scores for participants receiving CAB+RPV LA also reached the same levels at weeks 24 and 48 in both ATLAS and FLAIR, consistent with what was previously observed with the HIVTSQ. The lack of a statistically significant change from MBL in FLAIR was again mostly driven by high initial treatment acceptance and suggests that these newly treated patients are likely to find monthly LA treatment and daily oral treatment with a modern and well-established single-tablet regimen similarly acceptable. By contrast, those with more extensive experience of daily oral therapies over many years in ATLAS appeared to show a stronger predisposition in favor of LA therapy.

The results of the PIN questionnaire, assessing patient acceptability of injections, showed very similar attitudes towards IM administration between the two studies. Despite the common occurrence of ISRs, most participants indicated that the level of pain and ISRs was “very acceptable” 1 week after the initial LA injections, and scores improved significantly by 48 weeks of treatment. These trends are consistent with both the low discontinuation rate for ISRs and a declining incidence of ISRs across the maintenance phase of both studies [12, 13], similar to the time-dependent declines in ISRs seen with chronic LA parenteral treatment for other conditions, including infusions of ocrelizumab [34], rituximab [35], or ofatumumab [36] for multiple sclerosis, or IM injections of paliperidone [37] for treatment of schizophrenia.

In terms of general health outcomes, as measured by the SF-12 instrument, neither study showed statistically relevant changes from baseline in the physical or mental component subdomains for either oral or LA treatment. This is consistent with both the generally healthy baseline status of the two patient populations, and the noninferior efficacy of LA vs. oral treatment shown in both studies. The data also suggest that monthly injection therapy did not significantly alter participants’ general perceptions of their overall health status or functioning compared with oral therapy.

The results of the preference question were striking in both studies: 97% (ATLAS) and 99% (FLAIR) of responding participants preferred the LA regimen over prior oral therapy at week 48. Although willingness to participate in these studies assumes a predisposition to at least consider injectable therapy, the continued preference for the LA regimen is reassuring, suggesting that the LA regimen met participants’ expectations despite the challenges of monthly clinic visits and the prevalence of ISRs. Missing assessments, which contribute to the difference between ITT-E and per-responder analysis, are attributed to malfunctioning of PRO devices used for capturing data, scheduling of visits that fall within the dosing window but outside of the data collection window for PROs, and study withdrawals prior to week 48.

Specific factors driving the individual preferences in ATLAS may be multifaceted and vary between patients; however, convenience (53% of responders) and discretion (32%) were reported as the primary reasons for switching to LA therapy in the ATLAS study extension among those who had remained on oral treatment during the comparative treatment period.

Cognizance must be made of the limitations of these data in terms of the generalizability of the ATLAS and FLAIR study datasets to important sociodemographic groups chronically underrepresented in all HIV randomized trials—for whom the experience of LA vs. oral treatment may be different either in degree or in kind. Relevant sociodemographic factors such as employment, socioeconomic stratum, access to care, disability status, and drug use were not statistically captured in these studies. Also of note, even though both studies exceeded their female recruitment targets, which were set with high thresholds, this number was still low compared with the female proportion in the overall HIV population. While subgroup analyses may help provide additional clarity, for the more difficult-to-recruit groups it is likely that large observational cohorts will provide more robust future data on patient experience with LA treatment, including data on how well these trial results reflect the routine clinic situation. Also, the effect of longer (more than 48 weeks) LA treatment on PROs will be explored in forthcoming 96-week data from FLAIR, and from the results of the ATLAS-2M trial (NCT03299049) of 8-weekly vs. 4-weekly LA treatment, which included a high proportion of patients who rolled over from ATLAS after the 48-week primary analysis.

In addition, although treatment satisfaction has been shown to be positively correlated with adherence [38–40], the very high adherence rates in both studies do not allow for demonstration of potential adherence benefits with an LA treatment, with the additional caveat that adherence in a clinical trial may not always reflect that in the routine clinic. In ATLAS and FLAIR, 98% of LA dosing visits took place within the 7-day dosing window. Also, although pill counts were not provided, no protocol deviation was marked for patients in the CAR groups in terms of adherence to oral medication, signifying that total number of days with daily oral treatment interruptions did not exceed 10% per patient [41]. To address these evidence gaps, an ongoing clinical trial of CAB LA+RPV LA vs. oral treatment (LATITUDE; NCT03635788) is currently recruiting a more difficult-to-treat population with adherence challenges and a wider range of clinical, behavioral and demographic characteristics, where satisfaction with treatment and adherence with the LA regimen might be more pronounced.

While treatment differences for any individual PRO in an open-label trial may be subject to selection bias, there was a clear preference to continue with LA treatment in both the FLAIR and ATLAS patient groups randomized to receive it. In ATLAS, there was a clear advantage for LA treatment satisfaction and acceptance among patients who had previously received oral cART. Real-world uptake of LA treatment would in fact be driven by patient choice. The data suggest that availability of an alternative to daily oral treatment option will address the needs of a number of PLWHIV, including those who have challenges with remaining adherent to daily pills despite their efforts, medical conditions interfering with oral administration, those who wish to reduce the daily reminder of their HIV status, or those who simply prefer the convenience and flexibility associated with an LA treatment schedule.

In summary, these PRO analyses of participants in the FLAIR and ATLAS studies provide a patient-oriented perspective of the LA regimen. The results indicate a high degree of satisfaction, acceptance, tolerability, and preference for the LA regimen in multiple dimensions, supporting the therapeutic potential of monthly injectable LA therapy.

## Electronic supplementary material

Below is the link to the electronic supplementary material.

Supplementary file1 (TIF 829 kb) Supplementary Fig. 1. Change from baseline in individual HIVTSQs item score over 44 weeks in ATLAS. *BL* baseline, *CAB* cabotegravir, *CAR* current antiretroviral regimen, *HIVTSQs* HIV Treatment Satisfaction Questionnaire (status version), *LA* long-acting, *RPV* rilpivirine

Supplementary file2 (TIF 464 kb) Supplementary Fig. 2. PIN individual item domains and items in ATLAS. *ISR* injection site reaction, *PIN* Perception of Injection questionnaire, *SD* standard deviation
