# Supplementary figures and images for: Patient-Reported Outcomes in ATLAS and FLAIR Participants on Long-Acting Regimens of Cabotegravir and Rilpivirine Over 48 Weeks
Source: AIDS Behav. 2020 May 23;24(12):3533–44. doi: 10.1007/s10461-020-02929-8 (PMC7667137; doi:10.1007/s10461-020-02929-8)

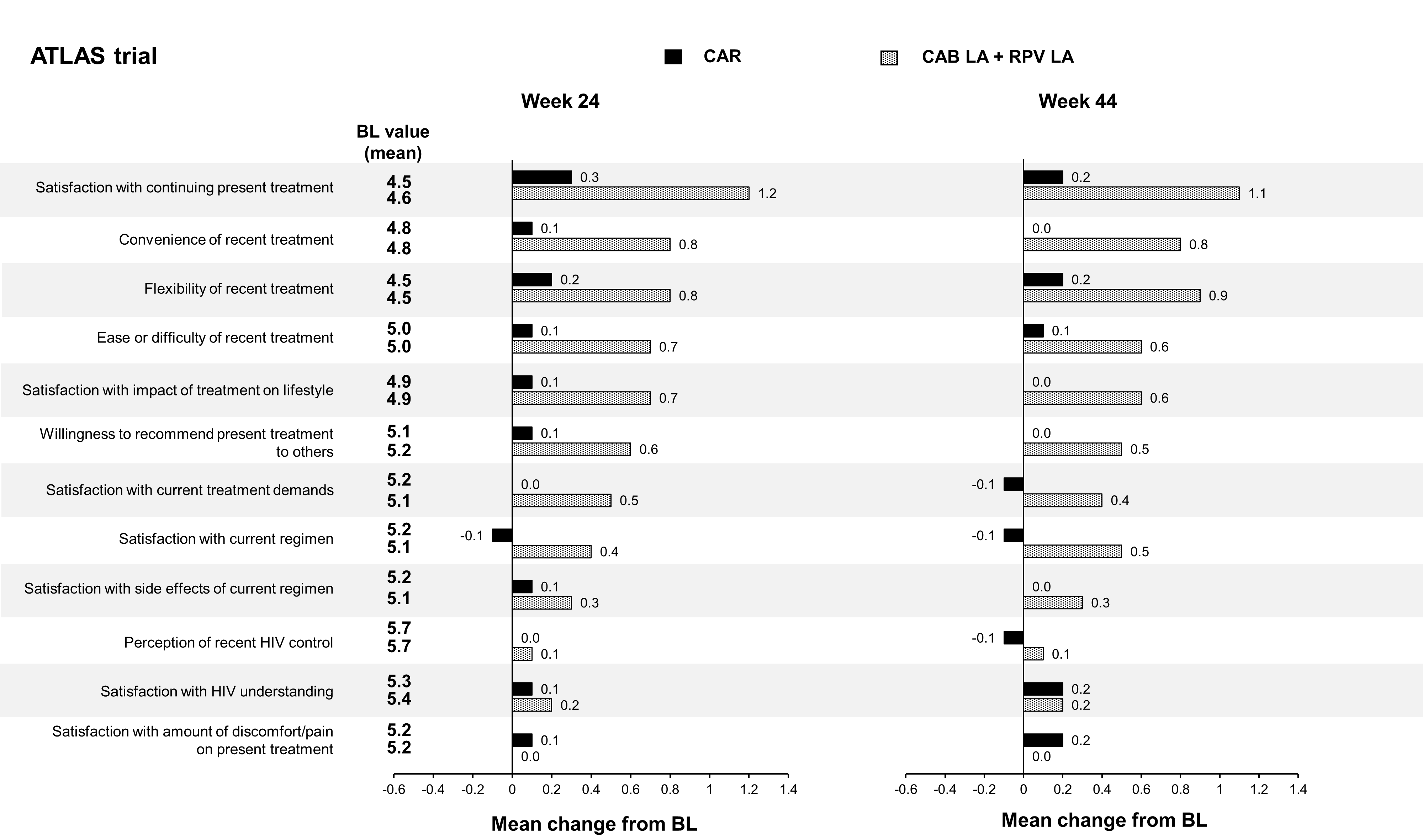

Supplement: Supplementary file 1 — Supplementary file1 (TIF 829 kb) Supplementary Fig. 1. Change from baseline in individual HIVTSQs item score over 44 weeks in ATLAS. BL baseline, CAB cabotegravir, CAR current antiretroviral regimen, HIVTSQs HIV Treatment Satisfaction Questionnaire (status version), LA long-acting, RPV rilpivirine [file 10461_2020_2929_MOESM1_ESM.tif]

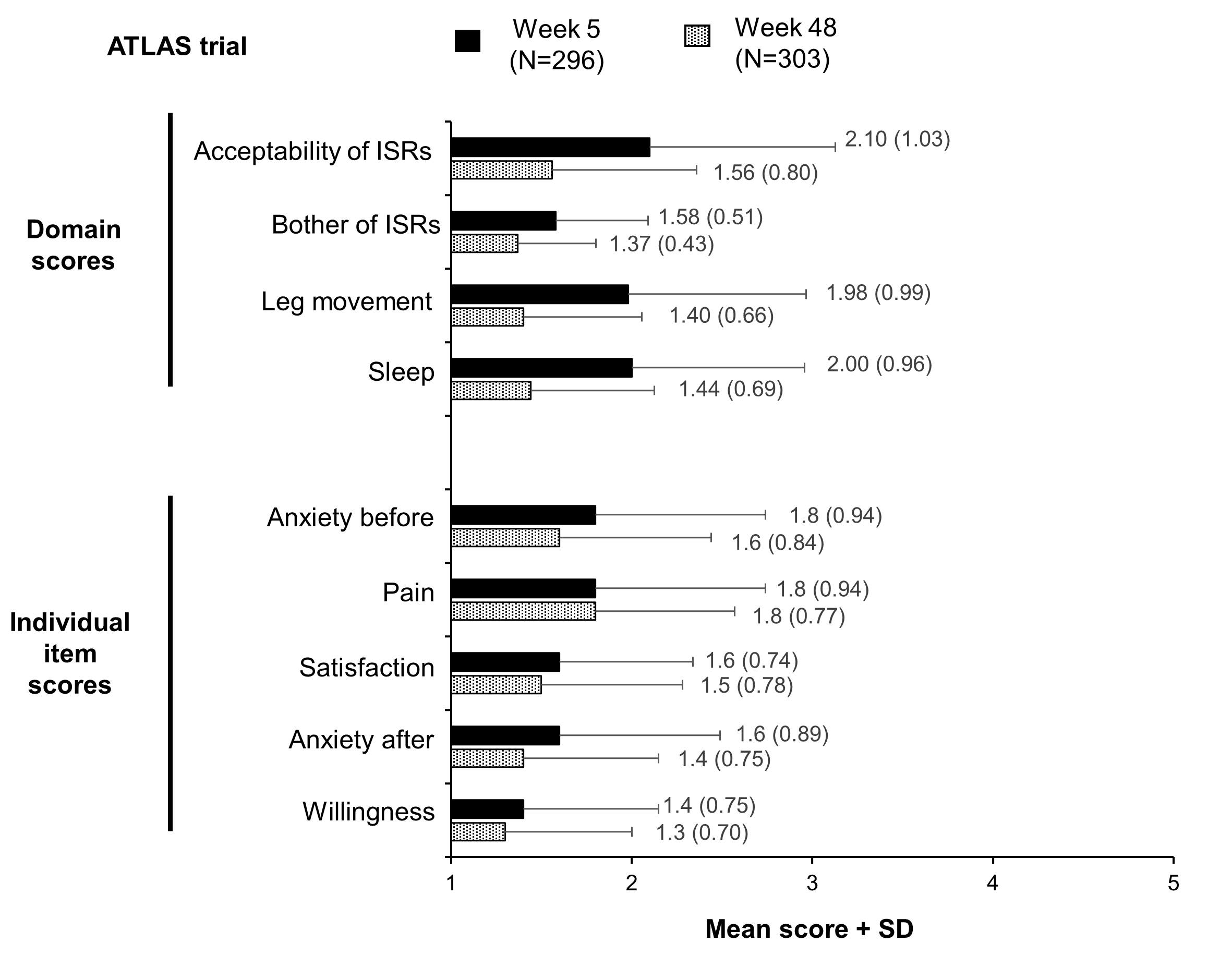

Supplement: Supplementary file 2 — Supplementary file2 (TIF 464 kb) Supplementary Fig. 2. PIN individual item domains and items in ATLAS. ISR injection site reaction, PIN Perception of Injection questionnaire, SD standard deviation [file 10461_2020_2929_MOESM2_ESM.tif]
